# Differential sympathetic outflow to adipose depots is required for visceral fat loss in response to calorie restriction

**DOI:** 10.1038/nutd.2017.13

**Published:** 2017-04-10

**Authors:** L M Sipe, C Yang, J Ephrem, E Garren, J Hirsh, C D Deppmann

**Affiliations:** 1 Department of Biology, University of Virginia, Charlottesville, VA, USA; 2 Department of Cell Biology, University of Virginia, Charlottesville, VA, USA; 3 Department of Biomedical Engineering, University of Virginia, Charlottesville, VA, USA; 4 Department of Neuroscience, University of Virginia, Charlottesville, VA, USA

## Abstract

The sympathetic nervous system (SNS) regulates energy homeostasis in part by governing fatty acid liberation from adipose tissue. We first examined whether SNS activity toward discrete adipose depots changes in response to a weight loss diet in mice. We found that SNS activity toward each adipose depot is unique in timing, pattern of activation, and habituation with the most dramatic contrast between visceral and subcutaneous adipose depots. Sympathetic drive toward visceral epididymal adipose is more than doubled early in weight loss and then suppressed later in the diet when weight loss plateaued. Coincident with the decline in SNS activity toward visceral adipose is an increase in activity toward subcutaneous depots indicating a switch in lipolytic sources. In response to calorie restriction, SNS activity toward retroperitoneal and brown adipose depots is unaffected. Finally, pharmacological blockage of sympathetic activity on adipose tissue using the β3-adrenergic receptor antagonist, SR59230a, suppressed loss of visceral adipose mass in response to diet. These findings indicate that SNS activity toward discrete adipose depots is dynamic and potentially hierarchical. This pattern of sympathetic activation is required for energy liberation and loss of adipose tissue in response to calorie-restricted diet.

## Introduction

Obesity has reached epidemic proportions in modern society. Pharmaceuticals have been developed that target endocrine pathways including leptin, ghrelin and insulin.^1, 2, 3^ Unfortunately, these drugs have proven largely ineffectual or unsafe in weight loss. The safest treatment for obesity remains dietary intervention. Although diet is the preferred method of remediation, there remains much that we don't know including: (1) What is the primary driver of adipose loss after dietary intervention? (2) Is adipose tissue lost evenly across the body in response to dietary intervention? Here, we seek to address these questions by examining an understudied mechanism governing energy liberation: the sympathetic nervous system (SNS).

Although obesity is characterized as an overall increase in adipose mass and lipid droplet storage, accumulation of specific fat depots are more directly associated with adverse health risks. In particular, the deposition of adipose mass in visceral depots correlates with pathologies, such as type II diabetes, cardiovascular diseases and some cancers.^4, 5, 6^ It has been suggested that proximity of these depots to internal organs and drainage of excess free fatty acids and inflammatory cytokines into the portal vein may contribute to such comorbidities.^7, 8, 9^ Alternatively, a link between upper body subcutaneous adipose and insulin resistance was elucidated.^10^ The reasons for these varied roles and associations between adipose and disease remain obscure, therefore it is necessary to identify points of regulation of individual depots.

The SNS is a key regulator of energy homeostasis through its control of heart rate, blood pressure and energy expenditure.^11, 12, 13^ Sympathetic nerve fibers innervate brown and white adipose depots to signal thermogenesis and energy liberation, respectively.^14^ Norepinephrine (NE) from sympathetic axon terminals binds the β3-adrenergic receptor (AR) on rodent white and brown adipose depots to induce lipase activation, which breakdown triglyceride stores.^15^ Free fatty acids and glycerol are then released into the bloodstream to be used as energy by other organs.^15, 16^ Lipolysis over long periods results in smaller adipocytes and decreased adipose mass.^15, 17^

Clues about SNS dynamics in energy homeostasis come from Bartness and colleagues, who challenged animals with fasting or cold exposure. When hamsters are fasted for 16 h, sympathetic outflow is increased dramatically to the subcutaneous inguinal depot and to a lesser extent to the visceral epididymal depot and not at all to the visceral retroperitoneal depot.^18, 19^ Likewise, hamsters and mice exposed to cold (5 °C) for 16 h display increased sympathetic drive to both brown and white adipose depots resulting in free fatty acids that can be converted to heat.^18, 20^ Taken together, these findings indicate that sympathetic outflow can differ between adipose tissues in response to a particular environmental challenge. These seminal studies indicate that not only does sympathetic outflow change in response to environmental challenge but it also appears to do so non-uniformly across adipose depots. Instead, depending on the environmental challenge, discrete fat pads preferentially receive increased sympathetic outflow. Although paradigm shifting, interpretation from these studies were somewhat limited because of the relatively short window of environmental challenge (16 h) examined. These studies were unable to ascertain whether discrete fat pads are preferentially and perhaps hierarchically used as lipolytic sources over sustained periods. Whether the SNS is required for diet-induced weight loss remains an open question. If this were the case, it would be important to define how SNS outflow to discreet fat pads is affected by prolonged time on diet. Such a longitudinal analysis would be particularly relevant to weight loss in humans, as increased sympathetic activity to adipose would drive lipolysis and reduce fat mass.

Here, we examine SNS activation toward discrete adipose depots in response to acute calorie restrictive diet over 12 days. We report a unique pattern of sympathetic activity to adipose depots throughout weight loss that dictates loss of discrete adipose depot mass. For example, sympathetic drive to visceral epididymal adipose is elevated early in weight loss and suppressed later in the diet. Coincident with the decline in sympathetic activity toward epididymal adipose, we observe an increase in sympathetic activity toward subcutaneous depots indicating a switch in lipolytic sources after extended time on diet. Using a β3-AR antagonist, we found that the sympathetic drive to adipose tissue is necessary for the loss of visceral adipose mass during calorie restriction (CR). Taken together this indicates that the SNS drives energy liberation from discrete adipose depots in a potentially hierarchical manner to manage overall energy homeostasis.

## Materials and Methods

### Mice and diets

All experiments were carried out in compliance with the Association for Assessment of Laboratory Animal Care policies and approved by the University of Virginia Animal Care and Use Committee (ACUC). The C57BL/6J (B6) mice were obtained from Jackson Laboratory (Bar Harbor, ME, USA). Mice used in all studies were adult male mice between 12 and 16 weeks of age and weighed at least 25 g. Mice were housed individually in a temperature-regulated room at 22 °C and kept on a 12 h light-dark cycle. Water was provided *ad libitum* (AL).

The standard chow diet (8604 Teklad rodent diet, Indianapolis, IN, USA) was provided to mice AL. The daily food intake of mice on standard chow was measured for 4 days and an average food intake was calculated. Mice on CR were given 75% of their daily standard chow intake. Calorie-restricted mice were fed daily within 1 h of the start of the dark cycle to ensure normal circadian feeding patterns. Owing to the ACUC regulations, mice on CR were not permitted to lose more than 20% of their starting body weight. All tissue and serum harvests were performed within 2 h of the dark cycle and before their daily allotment of food. Tissues were weighed immediately after dissection before being flash frozen in liquid nitrogen.

### NE turnover

Norepinephrine turnover (NETO) was measured using chemical inhibition of NE synthesis by alpha-methyl-*p*-tyrosine (AMPT) as described previously.^21^ The amount of NE in a tissue is a balance between production and degradation. NE is degraded rapidly after it is released into the synapse, therefore NE degradation is dependent and roughly equivalent to NE release.^22^ The total NE in adipose tissue before and after AMPT treatment was measured to obtain a rate of NE degradation independent of NE synthesis. The rate of NE degradation is multiplied by the total NE in the tissue as the final value of NETO.^18, 21, 23^ NETO is determined on a whole adipose depot basis to reflect the overall sympathetic drive and physiological impact for each tissue. The following two groups were used to determine the intra-animal decline of NE in tissues: (1) Mice not treated with AMPT and (2) mice treated with AMPT for 4 h. AMPT was administered by intraperitoneal injection at time 0 h (300 mg kg^−1^, 20 mg ml^−1^) and at time 2 h (150 mg kg^−1^, 20 mg ml^−1^). Adipose tissues were harvested and weighed 4 h after injection. NE was extracted from the tissue using a protocol adapted from Bartness and colleagues.^21^ In brief, an entire adipose depot was minced in 0.2 m percloric acid and 3 μg ml^−1^ Ascorbic acid. These samples were homogenized using the Bullet Blender (Next Advance, Averill Park, NY, USA) and the supernatants were column filtered before analyzing via HPLC.

The HPLC system consisted of a Jasco model PU-2080Plus isocratic pump and AS-950 Intelligent Autosampler (Jasco, Easton, MD, USA), an Antec Leyden Decade electrochemical detector (Antec Leyden, The Netherlands), pH 4.5 mobile phase with 4 mm decyl sulfonic acid/17% acetonitrile, as described.^24^

NETO was calculated by subtracting the NE content (ng NE/tissue) from control group from the AMPT treated group, to determine the rate of NE decline. Calculations were made according to the following formula: *k*=(lg[NE]^0^−lg[NE]^4^)/(0.434 × 4) and *K* =*k*[NE]^0^, where *k* is the constant rate of NE efflux, [NE]^0^ is the initial NE concentration, [NE]^4^ is the final NE concentration and *K*=NETO.^21^

### Measurement of serum metabolites

Blood was collected post euthanasia and centrifuged for 1 h 3000 *g* at 4 °C. Serum was collected then assayed for glycerol using the free glycerol reagent (Sigma, St Louis, MO, USA).

### Antagonist administration

Mice were either fed AL or placed on CR. Placebo mice were IP injected with saline. Treated mice were IP injected with 1 mg kg^−1^ SR59230a (Sigma) diluted in saline before being fed within 1 h before the start of the dark cycle.^25, 26^

### Statistical analysis

**Figure 1:**
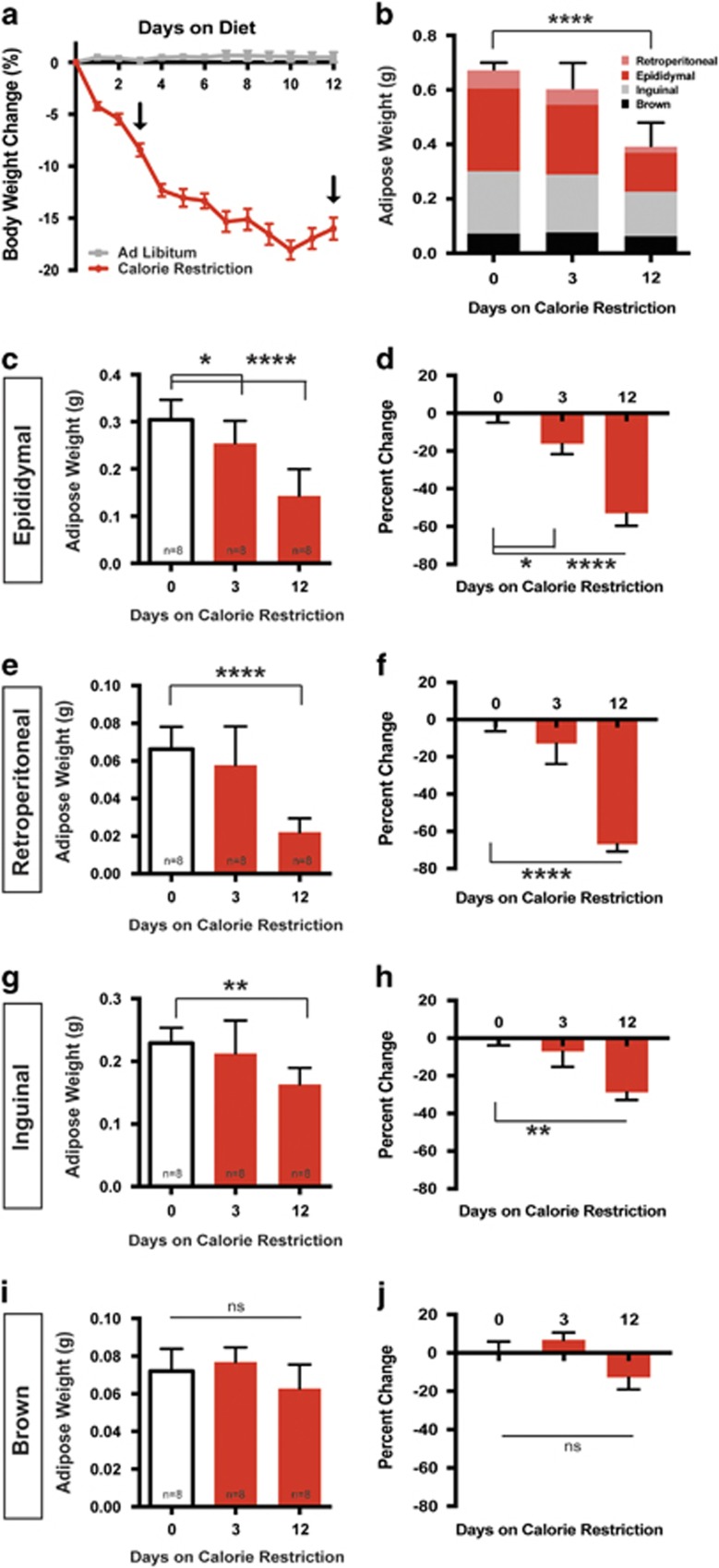
Selective loss of adipose mass in response to CR. (**a**) C57BL/6 male mice at least 12 weeks of age and 25 g were either fed AL or 75 % of the average daily standard chow intake. Mice were weighed daily and percent body weight change from starting body mass was calculated. Arrows mark early and late phases of weight loss (days 3 and 12) that are used in subsequent experiments. *n*=8 Two-way ANOVA (F_16,192_=79.47, *P*<0.0001). Tukey's multiple comparisons (*P<0.0001 day 0 compared to day 3 on CR, *P<0.0001 day 3 compared to day 12 on CR). (**b**) Total adipose weight of dissected adipose depots following 0, 3 or 12 days of CR. The contribution of individual depots to the total dissected adipose mass is shown. Pink=retroperitoneal, red= epididymal, gray=inguinal and black= intrascapular brown. One-way ANOVA (F_2,21_=24.42, *P*<0.0001) Tukey's multiple comparisons (*P*<0.0001 day 0 compared to day 12). (**c**) Dissected epididymal adipose mass after 0, 3 or 12 days of CR. One-way ANOVA (F_2,21_=22.54, *P*<0.0001) Tukey's multiple comparisons (*P=0.05 day 0 compared to day 3, ****P<0.0001 day 0 compared to day 12). (**d**) Percent change in epididymal adipose mass compared to the average of CR day 0. One-way ANOVA (F_2,21_=22.54, *P*<0.0001) Tukey's multiple comparisons (*P=0.05 day 0 compared to day 3, ****P<0.0001 day 0 compared to day 12). (**e**) Dissected retroperitoneal adipose mass after 0, 3 or 12 days of CR. One-way ANOVA (F_2,21_=21.54, *P*<0.0001) Tukey's multiple comparisons (***P=0.0001 day 0 compared to day 12). (**f**) Percent change in retroperitoneal adipose mass compared to the average of CR day 0. One-way ANOVA (F_2,21_=21.54, *P*<0.0001) Tukey's multiple comparisons (****P<0.0001 day 0 compared to day 12). (**g**) Dissected subcutaneous adipose mass after 0, 3 or 12 days of CR. One-way ANOVA (F_2,21_=6.95, *P*=0.0048) Tukey's multiple comparisons (**P=0.0048 day 0 compared to day 12). (**h**) Percent change in inguinal adipose mass compared to the average of CR day 0. **P=0.0034 day 0 compared to day 12. (**i**) Dissected intrascapular brown adipose mass after 0, 3 or 12 days of CR one-way ANOVA (F_2,21_=3.347, *P*=0.0547). (**j**) Percent change in intrascapular brown adipose mass compared to the average of CR one-way ANOVA (F_2,21_=3.347, *P*=0.0547). *N*=8 for all groups. Data shown as±s.e.m.

All statistical comparisons were performed using Prism 7 (GraphPad, San Diego, CA, USA). Specific statistical tests are explicitly stated in the figure legends. All data are expressed as the mean±s.e.m. Absolute adipose weights and NETO were analyzed with either a one-way ANOVA (Figure 1) or two-way ANOVA (Figure 4); both with Tukey's *post hoc* multiple comparisons. Percent changes of adipose weights were analyzed either a one-way ANOVA (Figure 1) or two-way ANOVA (Figure 4); instead with Dunnett's multiple comparisons against the control. No statistical methods were used to predetermine sample sizes, but our sample sizes are similar to those generally employed in the field. All data passed the D'Agostino and Pearson normality test. No blinding was performed for data analysis but randomization of mice was performed before placing them on diets.

## Results

### Adipose loss in response to CR occurs preferentially from visceral depots

We employed a CR model for dietary weight loss in mice over the course of 12 days. C57BL/6 adult male mice were limited to 75% of their individual standard chow intake. Mice on the calorie-restricted diet lose weight rapidly over the first 5 days, losing 13±2.4% of their starting body weight and by day 12 mice reach a loss of 16.9±2.8% (Figure 1a). Control mice that were fed AL maintained their weight over this 12 day period (Figure 1a). In this model of CR, we chose 3 and 12 days for subsequent analysis to reflect a period of early and late phase weight loss. Mice have significantly different body weights between the initial weight loss at day 3 and day 12 of CR.

We focused on the loss of adipose mass during calorie restriction. We first examined the summed mass of four adipose depots: epididymal, retroperitoneal, inguinal and brown. This total mass was not significantly reduced by day 3 compared to AL controls (Figure 1b). Instead, the loss of adipose mass is more gradual, with only significant loss of summed adipose mass observed after 12 days of CR (Figure 1b). The non-adipose loss that occurs by three days can be attributed to decreases in food intake, water retention and glycogen stores as has been reported in rodent, primate and human studies.^27, 28, 29^

We next sought to determine how individual adipose pads (that is, visceral or subcutaneous) lose mass as a function of time after CR. The only depot that displayed significant change by day 3 was epididymal adipose tissue, suggesting that this is an early source for lipolysis (Figures 1c and d). By day 12 of diet, both visceral depots examined decreased by half of their original weight. Epididymal adipose and retroperitoneal adipose mass reduced by 52.9±18.6% and 66.9±11.2%, respectively, compared to AL controls (Figures 1c–f). The subcutaneous inguinal adipose tissue displays a modest but significant decrease of 28.7±11.5% (Figures 1g and h). Intrascapular brown adipose displays no significant change in mass after 12 days on diet (Figures 1i and j).

### Dynamic sympathetic outflow to adipose depots in response to CR

We next examined whether changes in sympathetic outflow to particular adipose depots correspond with the loss of mass reported in Figure 1. Simply measuring the concentration of NE in a tissue is reflective of innervation density rather than release onto the tissue and in our experiments NE concentrations showed no differences over the course of the diet.^30^ Therefore, we turned to a classic method to measure of sympathetic activity, the NETO assay.^21, 31, 32, 33^ The amount of NE in a tissue is a balance between synthesis in the axon, release and degradation in the tissue. NE is degraded rapidly after it is released onto tissue, therefore, as suggested by Axelrod and colleagues, NE degradation is roughly equivalent to NE release.^22, 34^ We are able to assess NE turnover by isolating the rate of NE release and subsequent degradation using a chemical inhibitor of NE synthesis, AMPT.^21^ The rate of NE degradation over 4 h is multiplied by the total NE content of the adipose depot to obtain a final value of NETO to reflect the overall sympathetic drive toward each tissue.

**Figure 2:**
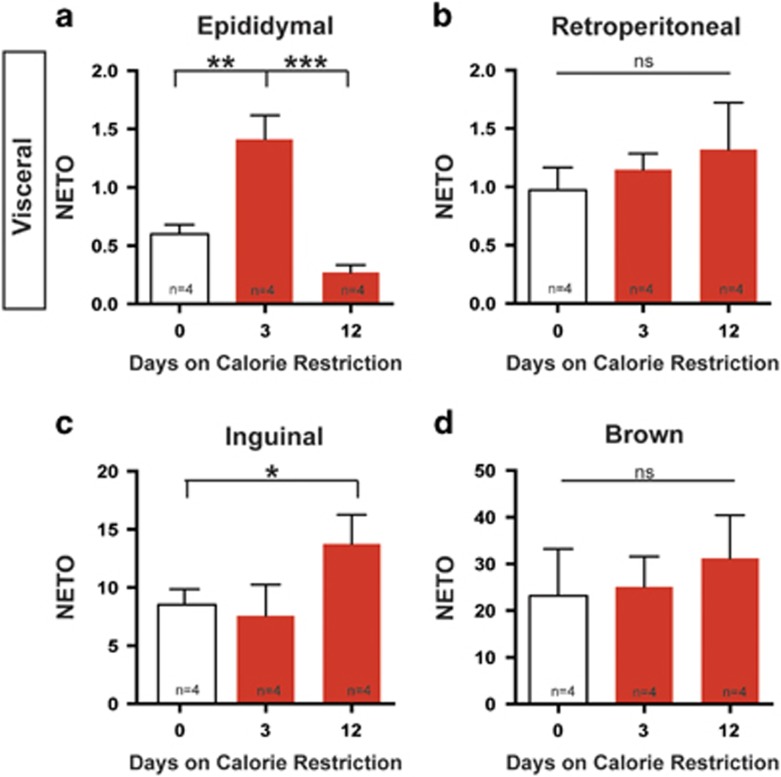
Non-uniform sympathetic activity to adipose depots after CR. (**a**) Norepinephrine turnover (NETO=(ng of NE in total adipose depot)^2^/hour) in epididymal adipose tissue after 0, 3 or 12 days of CR. One-way ANOVA (F_2,9_=19.48, *P*=0.0005) Tukey's multiple comparisons (*P=0.005 day 0 compared to day 3, *P=0.0005 day 3 verses day 12). (**b**) NETO in retroperitoneal adipose tissue after 0, 3 or 12 days of CR one-way ANOVA (F_2,9_=0.4715, *P*=0.6525). (**c**) NETO in inguinal adipose tissue after 0, 3 or 12 days of CR. One-way ANOVA (F_2,9_=7.296, *P*=0.0194) Tukey's multiple comparisons (*P=0.0374 day 0 compared to day 12). (**d**) NETO in brown adipose tissue after 0, 3 or 12 days of CR. One-way ANOVA (F_2,9_=0.9083, *P*=0.4372). *N*=4 for all groups. Data shown as±s.e.m.

We predicted that the timing of adipose mass loss of discrete depots would be matched by an elevation in sympathetic activity. Importantly, with enough time and sympathetic activity, we speculated that adipose depots would become depleted necessitating that sympathetic outflow to several of the tissues would be depressed at later time points. Indeed, the largest fat pad examined, the visceral epididymal adipose depot, displays a roughly 2.3-fold increase in NE turnover after three days of CR, which is consistent with the early loss of mass that we observe for this depot (Figures 1c and d; 2). This initial increase in NE turnover to epididymal adipose is decreased to baseline by day 12 of CR (Figure 2a). The impact of the sympathetic outflow on adipose mass would require prolonged lipolysis, therefore the significant decrease in adipose mass seen at day 12 is likely due to high sympathetic outflow after 3 days on diet (Figures 1c and d; 2a).

Importantly, we found that not all adipose depots followed the same pattern of NE turnover rate as the epididymal depot. The retroperitoneal adipose depot did not display a significant change in NE turnover rate in response to CR at either time point examined (Figure 2b). Despite this, retroperitoneal adipose still decreases in mass after CR (Figures 1e and f). Although this is the smallest of the white adipose depots examined, it suggests a mechanism for adipose mass loss that may be independent of changes to SNS outflow. Alternatively, increased sympathetic drive to retroperitoneal adipose may occur in between the time points analyzed.

Unlike the epididymal adipose where we observed initial increases in NE turnover, the inguinal adipose showed no change in NE turnover after 3 days (Figure 2c). Instead, increase in NE turnover to this depot occurred after 12 days of CR (Figure 2c). This delayed increase in SNS activity toward inguinal adipose corresponds to the modest reduction in adipose mass by 12 days (Figures 1g and h). Because ACUC regulations require that we remove animals from CR once animals have lost 20% of their body weight, we were unable to assess whether outflow to subcutaneous adipose would return to baseline with time.

In contrast to the dynamic sympathetic activity toward white adipose depots, brown adipose tissue does not show significant changes in sympathetic outflow or adipose mass (Figures 1i and j; 2d).

### Antagonizing sympathetic activity on adipose depots prevents diet-induced adipose loss

**Figure 3:**
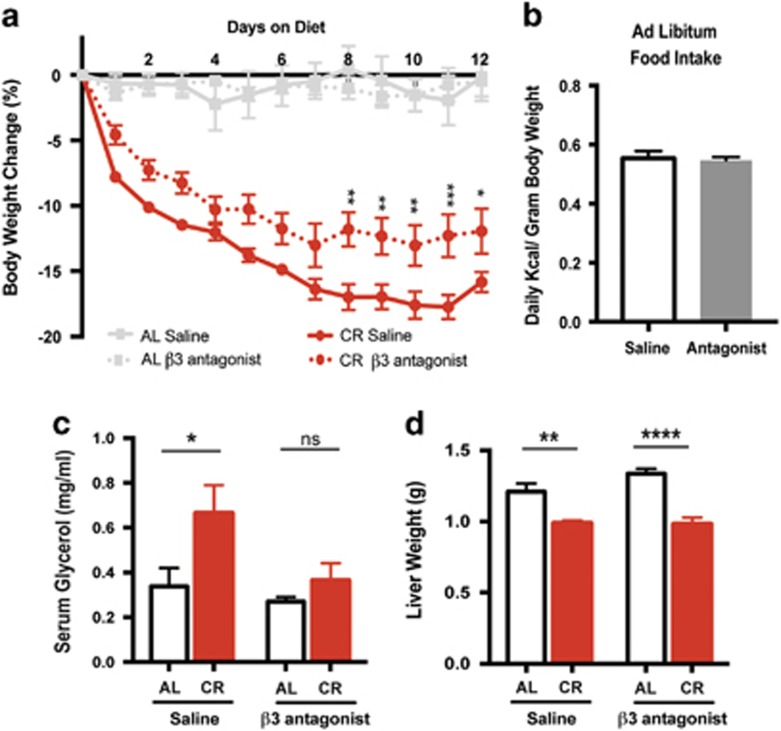
Inhibition of β3-AR signaling prevents body weight loss and lipolysis in response to CR. (**a**) C57BL/6 male mice at least 12 weeks of age and 25 g were either fed AL or only 75 % of their average daily standard chow intake (CR). Mice were injected IP daily with saline or SR59230a (1 mg kg^−1^) before feeding. Mice were weighed daily and percent body weight change from starting body mass was calculated. Two-way ANOVA (F_3,22_=48.14, *P*<0.0001) Tukey's multiple comparisons (*P<0.05 CR saline compared to CR SR59230A days 8–12). (**b**) Average daily food intake over 12 days by mice fed AL either injected IP with saline or SR59230a (1 mg kg^−1^). Two sample *t*-test (*P*=0.783). (**c**) Serum glycerol levels of mice after 12 days of AL feeding or CR either injected IP with saline or SR59230a (1 mg kg^−1^). *P=0.011 AL saline compared to CR saline, *P*=0.*956* AL SR59230a compared to CR SR59230a. (**d**) Dissected liver mass, weighed following 12 days of saline or SR59230a (1 mg kg^−1^) intraperitoneal injections while fed AL or on CR. **P=0.0083 AL SR59230a compared to CR SR59230a, ****P=0.0001 CR saline compared to CR SR59230a. Two-way ANOVA with Tukey's multiple comparisons. Data shown as±s.e.m. *N*=15 AL saline and CR saline *N=8* AL SR59230a *N*=13 CR SR59230a.

To determine the contribution of sympathetic activity to adipose tissue in dietary weight loss, we inhibited adipose specific adrenergic signaling using the β3-AR antagonist SR59230a.^25, 26, 35, 36^ The β3-AR is specifically expressed in both brown and white adipose tissue and is the primary receptor for NE on adipocytes in mice.^37^ Mice treated daily with SR59230a (1 mg kg^−1^) lost significantly less body weight on CR, evident by day 8 and through day 12, a time in which the majority of weight loss is from fat (Figures 1b and 3a). Mice fed AL had no change in body weight with daily treatment of SR59230a (Figure 3a). SR52930a treatment caused no change in AL food intake or consumption of the entire allotment of chow during CR (Figure 3b). Daily treatment with SR52930a inhibits CR induced lipolysis, which is predictive of a decrease in adipose mass loss (Figure 3c). Therefore, we conclude that the extent of weight loss that was inhibited can be primarily attributed to suppressed loss of adipose mass.

**Figure 4:**
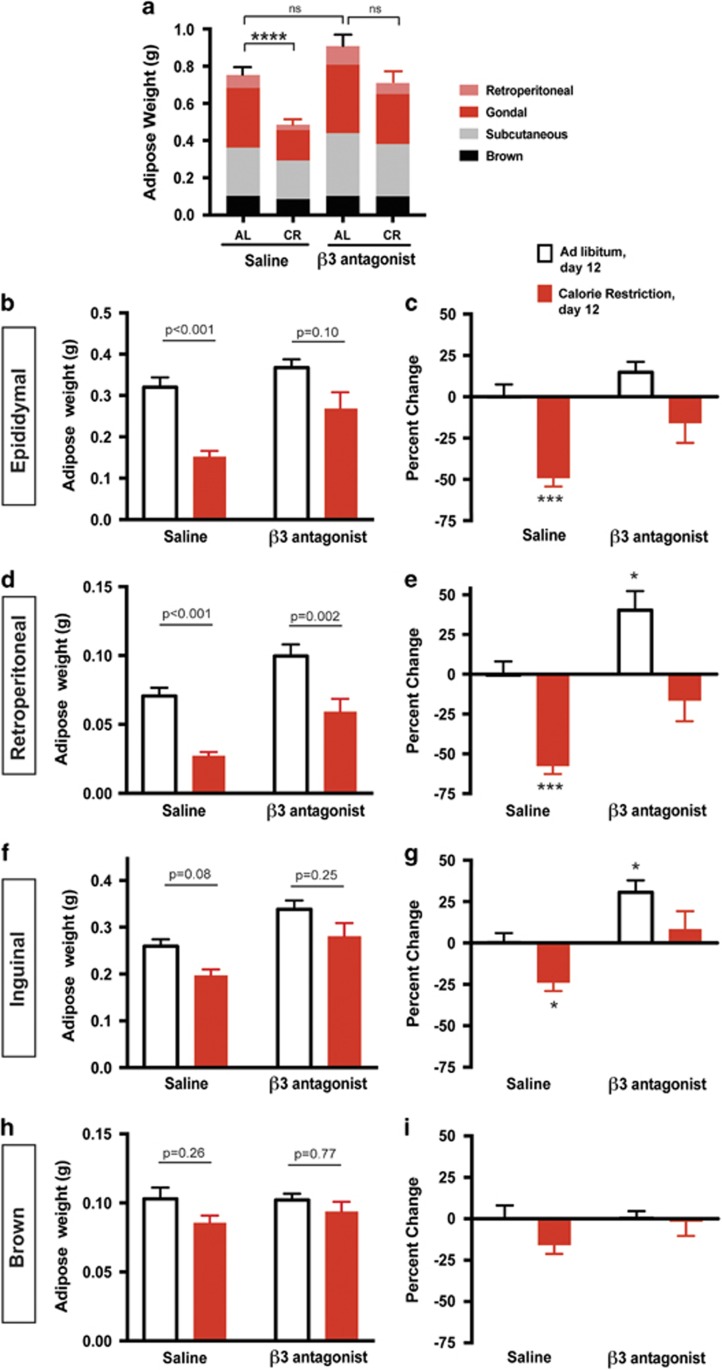
Inhibition of β3-adrenergic signaling in adipose tissue prevents loss of visceral and subcutaneous adipose mass. (**a**) Dissected adipose depots were weighed following 12 days of saline or SR59230a (1 mg kg^−1^) intraperitoneal injections while fed AL or on CR. The contribution of individual depots to the total adipose mass is shown. AL= *ad libitum*, CR=calorie restriction, pink=retroperitoneal, red=epididymal, gray=subcutaneous and black=brown. Two-way ANOVA (F_1,46_=9.396, *P*<0.0001) Tukey's multiple comparisons (****P<0.0001 AL saline compared to CR, *P*=0.32 AL saline compared to AL β3 antagonist, *P*=0.14 AL β3 antagonist compared to CR 3 antagonist, **P=0.0017 CR saline compared to CR β3 antagonist). (**b**) Epididymal adipose tissue. Two-way ANOVA (F_1,46_=21.88, *P*<0.0001) Tukey's multiple comparisons (****P<0.0001 AL saline compared to CR saline, *P*=0.1000 AL β3 antagonist compared to CR β3 antagonist, *P*=0.6526 AL saline compared to AL β3 antagonist, *P=0.0112 CR saline compared to CR β3 antagonist). (**c**) Percent change in epididymal adipose mass when compared to the average of AL fed saline treated mass. Two-way ANOVA (F_1,46_=9.396, *P*<0.0001) Dunnett's multiple comparisons (*P<0.001 AL saline compared to CR saline, *P*=0.5288 AL saline compared to AL β3 antagonist, *P*=0.3544 AL saline compared to CR β3 antagonist). (**d**) Retroperitoneal Adipose tissue two-way ANOVA (F_1,46_=36.18, *P*<0.0001) Tukey's multiple comparisons (****P<0.0001 AL saline compared to CR saline, **P=0.0026 AL β3 antagonist compared to CR β3 antagonist, *P=0.0381 AL saline compared to AL β3 antagonist, **P=0.0060 CR saline compared to CR β3 antagonist). (**e**) Percent change in retroperitoneal adipose mass when compared to the average of AL fed saline treated mass. Two-way ANOVA (F_1,46_=33.21, *P*<0.0001) Dunnett's multiple comparisons (*P<0.0001 AL saline compared to CR saline, *P*=0.0239 AL saline compared to AL β3 antagonist, *P*=0.4696AL saline compared to CR β3 antagonist). (**f**) Inguinal adipose tissue two-way ANOVA (F_1,46_=8.93, *P*=0.0045) Tukey's multiple comparisons (*P*=0.0838 AL saline compared to CR saline, *P*=0.2587 AL β3 antagonist compared to CR β3 antagonist, *P*=0.0558 AL saline compared to AL β3 antagonist, *P=0.0144 CR saline compared to CR β3 antagonist). (**g**) Percent change in inguinal adipose mass when compared to the average of AL fed saline treated mass. Two-way ANOVA (F_1,46_=8.934, *P*=0.0045) Dunnett's multiple comparisons (*P=0.048 AL saline compared to CR saline, *P*=0.0318 AL saline compared to AL β3 antagonist, *P*=0.7517 AL saline compared to CR β3 antagonist). (**h**) Brown adipose tissue. Two-way ANOVA (F_1,46_=4.085, *P*=0.0537). (**i**) Percent change in brown adipose mass when compared to the average of AL fed saline treated mass. Two-way ANOVA (F_1,46_=4.08, *P*=0.0537). Data shown as±s.e.m. *N*=15 AL saline and CR saline *N=8* AL SR59230a, *N*=13 CR SR59230a.

We next sought to test the effect of SR52930a on adipose mass by examining summed adipose loss as described in Figure 1a. Although SR59230a treated mice fed AL displayed no significant change in total adipose mass (Figure 4a), we did observe increases in individual depots retroperitoneal and inguinal weights (Figures 4e and g). During AL feeding, the balance between energy storage and liberation is skewed toward energy storage. On CR, the system shifts toward energy liberation, which these data suggest is primarily though the SNS. Nevertheless, the most profound effect of SR59230a is on diet-induced fat loss. Consistent with observations in Figure 1, mice treated with saline had decreased total adipose mass after 12 days on CR (Figure 4a). However when treated with SR59230a, mice no longer lost significant total adipose mass after CR (Figure 4a). Treatment with SR59230a has the most dramatic impact on visceral adipose depots. Consistent with observations in Figure 1, epididymal adipose under saline treatment displayed a 49.2±5% reduction in mass after 12 days of CR (Figures 4b and c). Interestingly, epididymal adipose mass from calorie-restricted SR59230a treated mice decreased by only 16±11.8%, which is statistically insignificant compared to saline treated AL fed mice (Figures 4b and c).

Unlike the epididymal tissue, SR59230a affected the retroperitoneal adipose mass in AL and calorie-restricted conditions. *Ad libitum* fed mice treated with SR59230a displayed a roughly 40% increase in retroperitoneal adipose mass (Figures 4d and e). Under CR, SR59230a treated mice only lost 15% of retroperitoneal adipose mass compared to the 60% under saline treatment (Figures 4d and e). In this experiment, inguinal adipose mass still decreased by 30%, but did not reach the significance observed in Figure 1. This relatively small change (compared to 60–80% in visceral depots) as well as the additional statistical stringency added by the two-way ANOVA could account for the discrepancy in statistical significance between Figure 1g and 2f. In addition, when represented as percent change and analyzed with Dunnett's multiple comparisons there is a significant decline in adipose mass after CR. Nevertheless, the inguinal depot did not show significant weight loss after antagonist treatment, due to the role of sympathetic drive in weight loss (Figures 2f and g). Brown adipose mass displayed no change after CR and likewise SR59230a treatment had no effect on their mass (Figures 2h and i). Taken together, these data suggest that the visceral depots are uniquely sensitive to sympathetic activity both with respect to energy storage and liberation.

## Discussion

Here, we demonstrate that sympathetic drive regulates preferential loss of visceral adipose mass after acute calorie restriction. Clinical reports in humans show visceral adipose tissue is preferentially lost during acute weight loss diets.^38, 39^ We suggest that the mechanism underlying this phenomenon is via selective sympathetic drive to visceral depots during calorie restriction. This also suggests that the SNS may be a relevant target for human obesity since excess visceral adipose is linked to several comorbidities including Type II diabetes, cardiovascular disease and some cancers.^8^

In response to diet, we found that not only can the dynamics of sympathetic drive differ between organs but also within the same organ. A similar conclusion was made using Siberian hamsters, when, in response to fasting, SNS activity toward visceral epididymal depot was elevated, whereas activity remained unchanged in both inguinal white adipose and brown adipose tissue.^18^

The differences in sympathetic outflow dynamics in response to calorie restriction suggest that adipose depots may be used in a hierarchical manner as lipolytic sources and may represent a general logic for energy homeostasis. In our model of dietary weight loss we observed an initial increase of sympathetic drive to the visceral epididymal adipose depot. This was the only depot to significantly decrease in mass after 3 days of calorie restriction (Figure 1c). This suggests that epididymal depots are an early source of lipolysis in conditions of energy deprivation.

**Figure 5:**
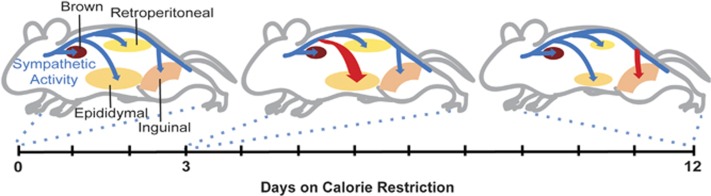
Model of hierarchical dynamics of sympathetic outflow during calorie restriction. The SNS (blue) innervates the visceral adipose depots epididymal and retroperitoneal, the inguinal depot and the intrascapular brown adipose depot. After 3 days of calorie restriction sympathetic outflow is increased to the epididymal adipose tissue (red arrow). The sympathetic activity causes the epididymal adipose depot to shrink significantly by day 3 and reduce by half of the original size at day 12. By day 12 of CR, sympathetic outflow to epididymal adipose returns to normal, whereas the activity to subcutaneous adipose increases. The sympathetic drive to retroperitoneal adipose does change as a function of diet, however the depot reduces in mass by day 12 perhaps by SNS independent mechanisms. The brown adipose depot sees no changes in sympathetic drive or adipose mass during the 12 days of calorie restriction.

Sympathetic drive to adipose tissue may define the preference or hierarchy of lipolytic sources to be used for energy. Indeed, we observe elevated SNS activity first toward visceral depots during calorie restriction and then switching to subcutaneous depots later in the diet. Whether this switch is dependent of depletion of visceral stores remains an open question. After 12 days of calorie restriction the sympathetic drive to epididymal adipose tissue decreases below baseline, whereas the drive to inguinal adipose becomes significantly elevated (Figures 3a and c; 5).

It is important to note, that tissues are not uniformly sensitive to NE due to regulation of ARs or downstream signaling.^40^ In fact, subcutaneous adipose tissue has increased expression of Gαi coupled alpha-ARs relative to visceral depots, which would inhibit the signals for lipolysis and resist loss of adipose mass.^41, 42^ This may explain why we observe only a slight decrease in subcutaneous adipose mass by twelve days of calorie restriction, despite observing elevated NE turnover to this depot (Figures 1b and e; 2c). It will be interesting in the future to examine the dynamics of AR expression in this and other adipose depots as a function of time on calorie restriction.

Diet-induced changes in sympathetic activity appear to be required for dietary fat loss. When mice were treated with a β3 antagonist, the visceral adipose depots no longer significantly decreased in mass after calorie restriction (Figures 2a–e). This finding has clinical relevance, because β-AR blockers are a common treatment for obesity-induced hypertension. Consistent with our findings, patients on some β-AR blockers gained body weight in the first 2 years.^43, 44^

Not all adipose loss required dynamic SNS activity. The retroperitoneal depot lost mass in a similar fashion to epididymal adipose, however there was no significant increase in NE turnover as a function of time on diet (Figures 1g and h; 3b). Interestingly, the β3-AR inhibitor, SR52930a, protects this adipose depot from diet-induced loss of mass and increased the mass while fed AL. This indicates that although SNS activity wasn't changed in the time points assessed, it may still be involved in adipose maintenance and energy storage within the retroperitoneal depot.

The mechanisms underlying increased or decreased sympathetic drive to particular adipose depots are not well understood. As parasympathetic nerves do not innervate any adipose depot, the sympathetic drive to adipose is without the regulatory balance typical of the autonomic nervous system.^45, 46^ There are several possible mechanisms by which autonomic drive to adipose might be tuned: (1) A sensory feedback mechanism, whereby axons respond to lipolysis breakdown products, adipose mass, lipid stores or leptin levels to control adipose specific sympathetic circuits^47, 48, 49, 50, 51^ A circuit whereby sensory afferents directly or indirectly feedback onto sympathetic outflow is an attractive model for how adipose tissue may tune homeostatic energy liberation.^11^ Indeed, points of sympathetic and sensory interaction have recently been identified for brown adipose tissue, such as the raphe pallidus nucleus, nucleus of the solitary tract, periaqueductal gray, hypothalamic paraventricular nucleus and medial preoptic area.^52^ (2) This may also be explained by an endocrine feedback loop, where adipose-derived signals travel through the bloodstream directly to the CNS or perhaps postganglionic neurons to regulate activity. We do not favor this model because it is difficult to envision a mechanism whereby activity to particular depots is selectively regulated.

Our findings have implication for dietary intervention of obesity. Of note, elevated muscle sympathetic tone has been linked to increased visceral adiposity in obese patients, independent of total adipose, subcutaneous adipose or age.^53^ Our research has provided further evidence that visceral adipose and sympathetic drive are tightly linked. In obese settings, targeting the sympathetic drive to adipose might be detrimental as increased serum-free fatty acids can lead to insulin resistance, fatty liver and glucose intolerance.^54, 55^

We have determined the dynamics of the SNS activity toward visceral, subcutaneous and brown adipose tissue. Our findings suggest that sympathetic drive defines preferential changes in adipose depots, which represents a critical therapeutic target. In the future, it will be important to examine the dynamics of the SNS in other dietary challenges (that is, high fat or ketogenic) as well as potential changes in sex and age. The potentially hierarchical dynamics of the SNS activity in response to persistent dietary challenge represent a novel mechanism for body weight maintenance and energy homeostasis.
